# The effect of an acute antioxidant supplementation compared with placebo on performance and hormonal response during a high volume resistance training session

**DOI:** 10.1186/1550-2783-11-10

**Published:** 2014-03-21

**Authors:** James Ackerman, Tom Clifford, Lars R McNaughton, David J Bentley

**Affiliations:** 1 Health and Exercise, School of Medical Sciences, University of New South Wales, Sydney, Australia; 2 Department of Exercise Science, University of Portsmouth, Portsmouth, UK; 3 Department of Sport and Physical Activity, Edge Hill University, Ormskirk, UK; 4 Human Exercise Performance Laboratory, School of Medical Sciences, University of Adelaide, Adelaide 5005, South Australia

**Keywords:** Fatigue, Ergogenic, Response, Anabolic, Exercise

## Abstract

Antioxidant supplementation is known to increase human endogenous antioxidant (AOX) capacity providing a means of blunting exercise induced reactive oxygen species (ROS). The purpose of this study was to compare the effects of a single acute dose of an AOX (vs blinded placebo) on muscle contractile performance and hormonal responses to a single bout of lower limb ‘hypertrophic’ resistance training (RT). Fifteen resistance trained subjects (age 23 ± 4 years: body mass 86 ± 6 kg) volunteered to participate in the study. Each subject attended the laboratory on three occasions, firstly to determine three repetition maximum (3-RM) isotonic strength in the back squat and perform a familiarisation of the experimental task. On the second/third visits subjects completed the hypertrophic training session (HTS) which consisted of six sets of 10 repetitions of 70% of a predicted 1 RM load (kg). Four hours prior to the HTS the subjects consumed 2 ml#x2219;kg^−1^ total body mass of either the placebo mixture or AOX supplement in a randomised order. Work completed during the strength training session was completed with equipment that had an integrated linear force transducer (Gymaware system, Kinetic Performance Technology, Canberra, Australia). During the placebo trials concentric mean power significantly (*p* < 0.05) decreased from sets 1–6. Accumulated power output during the AOX HTS was 6746 ± 5.9 W which was significantly greater compared to the placebo HTS of 6493 ± 17.1 W (*p* < 0.05, ES’r = 0.99). Plasma growth hormone (GH) concentration was significantly less immediately following AOX supplementation (6.65 ± 1.84 vs 16.08 ± 2.78 ng#x2219;ml^−1^; *p* < 0.05, ES’r = 0.89). This study demonstrates ingestion of an AOX cocktail prior to a single bout of resistance training improved muscle contractile performance and modulated the GH response following completion of the resistance exercise. Future studies should explore the mechanisms associated with the performance modification and specific muscle adaptations to AOX supplementation in conjunction with heavy RT.

## Background

Reactive oxygen species (ROS) have been implicated as one of the causes of skeletal muscle fatigue during both aerobic and anaerobic exercise [1]. Although small increases in exercise induced ROS are important for stimulating cellular growth and maximising muscular force production [2,3], excessive accumulation leads to a pro-oxidant environment which can damage DNA, lipid and protein membranes [4,5]. Cellular damage may also impair cross-bridge cycling during skeletal muscle contraction and accelerate the onset of fatigue [2,6,7]. This is supported by previous work suggesting that a bout of resistance training induces an excessive increase in ROS production which could be implicated in the reduction in skeletal muscle force generating capacity observed during exercise [4,8,9].

To maximise gains in muscular hypertrophy an RT session would typically involve exercising at a moderate intensity, defined as lifting a load between 65-85% of an individual’s one repetition maximum (RM), and using a high volume, typically 3–6 sets of 6–15 repetitions of the exercise [10]. Goldfarb and colleagues [8] found significant increases in the plasma ROS markers malondialdehyde (MDH) and protein carbonyls (PC) following arm flexor exercise involving four sets of a 12 repetition maximum (RM) load. Similar results have also been found for lower body resistance exercise where plasma measures of oxidised gluthanione (GSSG) and protein oxidation were elevated following 30 min of sub-maximal squatting exercise [4]. The primary cause of RT induced oxidative damage appears to result from increased xanthine and nicotinamide adenine dinucleotide phosphate (NADPH) oxidase production, together with ischemia–reperfusion which results in an increase in xanthine oxidase (XO) and peroxynitrite [9,11,12]. Xanthine oxidase production appears to be a primary cause of oxidative stress during vigorous exercise [13], and plasma concentrations have been shown to increase dramatically after performing an RT session [14]. Other sources such as a decrease in intracellular pH, lactate accumulation and sarcomere disruption can also contribute to RT induced ROS production [4,2].

It has been suggested that a supplementation regime of antioxidants could reinforce the body’s endogenous antioxidant system providing a means of blunting exercise induced ROS molecules [15,16]. Several studies have demonstrated that AOX supplementation can minimise damage to cellular structures caused by RT [8,17] and also help maintain muscular force [18] during isometric maximal contractions. However, there are also a number of studies that have found no benefit of AOX supplementation on markers of oxidative stress or performance [19-21]. Differing exercise protocols, subjects and types/amounts of AOX supplements used, have been suggested as the cause of the inconsistency between findings [21]. It appears that RT protocols employing a higher volume and intensity invokes the greatest oxidative stress response, while there is some support for the effectiveness of Vitamins C and E and flavonoid supplements at attenuating acute muscle injury in untrained individuals [21].

Most AOX studies have focused on the effects of vitamin C and/or E supplementation to attenuate the oxidative stress caused by RT [8,18-20]. There has been little focus on plant polyphenols, which have potent antioxidants qualities [22,23]. Pycnogenol (PYC) is a particularly effective antioxidant polyphenol, comprised of several proanthocyanidins and phenolic acids and has been shown to blunt elevated ROS [24,25], increase growth hormone (GH) secretion [26] and stimulate muscle blood flow [27]. It has also previously been shown that the supplement Lactaway©, containing PYC, acutely improves endurance cycle performance without improving AOX capacity [28,29]. There are no studies that have yet assessed the effects of Lactaway© containing PYC on RT performance and the associated bio-molecular responses. Hence, this study aimed to assess the effect of a PYC mixture, on performance during lower limb ‘hypertrophic’ RT (HRT) and the resulting acute endocrine, physiological and oxidative stress response.

## Methods

### Subjects

Fifteen healthy subjects volunteered to participate in the study (age 23 ± 4 yr: body mass 86 ± 6 kg: height 179.4 ± 6.1 cm). Each subject had been resistance training for a minimum of 2 yr prior to recruitment for the study. All the subjects were familiar with the back squat exercise (BS) and could perform the activity satisfactorily from a technique perspective. Assessment was carried out by the primary researcher who was a certified strength and conditioning coach. Each subject completed a consent form and pre activity screening questionnaire to identify any musculoskeletal and orthopaedic problems that could affect performance of the exercise. The study gained approval from the University of New South Wales, Human Research Ethics Committee.

### Study design

A double blind repeated measures design was employed where the subjects ingested either the AOX treatment or a placebo version prior to completing the training session. In the 48 h leading up to these sessions the subjects were instructed to refrain from intense physical exercise in order to eliminate residual fatigue. The supplements were provided using a randomized and counterbalanced design. The subjects visited the laboratory on three occasions, firstly to record their physical characteristics and determine their 3 RM BS which was used to predict 1RM strength [1.06 × 3RM (kg) [30]]. On their second and third visits subjects completed the hypertrophic training session (HTS) which consisted of six sets of 70% of 1RM. The BS was performed with an Olympic barbell using a power rack (Body Maker, Nantong, China). The depth of the squat was controlled by placing the safety spot inserts of the squat rack device just below of the level the barbell when subject’s thighs were parallel to the ground. This acted as a feedback mechanism for the participant and researcher but participants were asked to refrain from “bouncing” on the parallel bars. The subjects were instructed to refrain from alcohol, foods with high AOX capacity and caffeine for 24 h prior to HTS. This information was in a document which was read to each subject prior to commencement of participation in the study. Subjects recorded their diet and were asked to replicate the same dietary intake 24 hrs prior to each session.

### Preliminary measures and familiarisation

On the subjects’ first visit, their body mass (kg) was measured using a balance beam (Weylux, England) and height (cm) with a stadiometer (Holtain Ltd). Subjects then undertook a warm on up on a cycle ergometer (Schroberer Rad MeBtechnik (SRM), Weldorf, Germany), cycling at 1 watt·kgˉ^1^ for five min. The determination of the 3RM was followed according to methods previously described [31]. Briefly, it required approximately four to six sets to determine the 3RM with progressively heavier loads per set. Three min rest was allowed between each set and the 3RM was determined as the load lifted three times and when no extra weight could be added. The 3RM was used to predict 1RM for each participant. After a five min break a squat session of 10 repetitions at 70% 1RM load for five sets was performed.

### Experimental procedures and supplements

Four hours prior to the HTS the subjects consumed 2 ml#x2219;kg^−1^ body mass of either the placebo mixture or AOX supplement [Lactaway©, Away Australia Pty Ltd, Sydney, Australia] containing 2.4 g#x2219;L of PYC in a randomised order. The placebo and AOX mixtures tasted and appeared the same. The participants and researchers were not aware of which substance was supplement or placebo until after the completion of the study when details were released by an independent person. The placebo mixture contained the exact energy content (315 KJ) and constituents (pineapple pulp, molasses, sodium chloride, flavour, steviol glycosides, sodium benzoate, potassium sorbate) as the Lactaway© mixture (315 KJ) but it did not include any of the active AOX ingredient PYC.

After a brief cycling warm up, the subjects completed a warm up set consisting of 10 repetitions at 50% of the actual load to be used during the work sets. After a two min rest period the subjects performed the second warm up set at 80% of the load to be used during the work sets. After a three min rest period, subjects completed six sets, separated by 2 min rest periods. The subjects were instructed to lower the barbell under control (eccentric) and then verbally encouraged to “drive” the barbell upwards in as short as time possible (concentric). The squat training session lasted ~18 min. After the completion of each set the subjects were also asked their rate of perceived exertion (RPE) using the Borg scale [32]. Five microliter (μL) finger tip capillary blood samples were collected under standard aseptic procedures before, immediately after and twenty min post-exercise to analyse blood lactate (LT 1710 Lactate Pro, KDK Corporation, Shiga, Japan).

An integrated linear force transducer (Gymaware system, Kinetic Performance Technology, Canberra, Australia) was used to determine barbell displacement for each repetition and set completed. This system allows for the determination of concentric mean power (W), and concentric velocity (m·s) to be determined. The system was set up according to the manufacturer’s guidelines and has been shown to provide a reliable (Coefficients of variation (CV) = 3.3%) and valid estimate of power during resistance training [33].

### Blood collection and analysis

Venous blood was withdrawn via venepuncture before, immediately after and twenty min after the HTS. Blood was collected from a vein in the cubital fossa in ethylenediaminetetraacetic acid (EDTA) (10 ml tube) vacutainers (BD367863, NJ, USA). The samples were then centrifuged at 3000 rpm for 10 min, at 4°C. The plasma top layer was placed into Eppendorf tubes (Oldenburg, Germany) and snapped frozen and stored at −80°C until analysis.

Plasma GH, an indicator of the anabolic hormonal milieu during RT [34] was determined pre-exercise, immediately post-exercise and 20 min post-exercise. Plasma GH was assayed by a radio-immunoassay using a commercially available kit (human growth hormone ELISA DSL-10-1900, Diagnostic Systems Laboratories, Webster, USA). The assay was performed in duplicate as per the instructions from DSL and determined the levels of the 22 kDa GH isoform. The CV was less than 7% for the assays and the limit detection was 0.03 ng/ml.

Plasma cortisol (CORT) was measured as an indicator of the catabolic hormonal environment during RT [34], and was determined by a radio-immunoassay using a commercially available kit (cortisol ELISA DSL-10-2000, Diagnostic Systems Laboratories, Webster, USA). Plasma CORT was assayed pre-exercise, immediately post-exercise and 20 min post-exercise. The assay was performed in duplicate as per the instructions from DSL and the CV was less than 10%.

Xanthine oxidase (XO) was measured because it is involved in free radical production and its elevation contributes to oxidative stress [12,13]. The XO was assayed in duplicate using a commercially available kit (Invitrogen, Carlsbad, California, USA). Plasma was assayed pre-exercise and immediately post-exercise. The XO stock solution was used to construct a standard curve. The standards and serum were pipetted into a high binding enzyme immunoassay (Caymen Chemical Co. Ann Arbor, MI USA) 96 well plate. The plasma samples were diluted 1 fold by the placement of a buffer solution, and the XO reaction was started when a composition of amplex red, horseradish peroxidase, hypoxanthine and buffer solution was added to each well. The plate was incubated at 37°C for 30 min and the absorbance was read at 550 nm using a PolarStar Galaxy plate reader (BMG Laboratory Technologies, Offenburg, Germany).

### Statistical analysis

A two way repeated measures analysis of variance (ANOVA) was used to evaluate changes over time and condition for power and velocity along with lactate, RPE, GH, CORT and XO. If a significant F value was achieved the Bonferroni post hoc test was performed. The level of significance was set at p ≤ 0.05. All data was analysed using SPSS for Windows version 16. Data are presented as mean ± standard error of the mean (SEM). Where relevant effect size ratios (ES’r) were calculated using Cohens *d*[35]. An ES’r of ≥0.5 was considered to display a moderate effect and ≥0.8 a large effect.

## Results

The pre to post HTS, blood lactate concentrations (B_lac_) increased significantly after both AOX supplementation; 1.23 ± 0.08 to 7.68 ± 3.01 mmol^.^l^−1^ (*p* < 0.05) and placebo supplementation; 1.79 ± 0.30 mmol^.^l^−1^ to 8.11 ± 2.98 mmol^.^l^−1^ (*p* < 0.05). Blood lactate continued to be significantly elevated twenty min post-exercise for both groups, but there was no significant difference in B_lac_ levels between the two conditions at any time point (*p* > 0.05). The RPE was significantly increased in both groups for sets three to six compared to set one. There were however no significant differences in RPE between the AOX and placebo conditions at any point during the HTS (*p* < 0.05).

**Figure 1:**
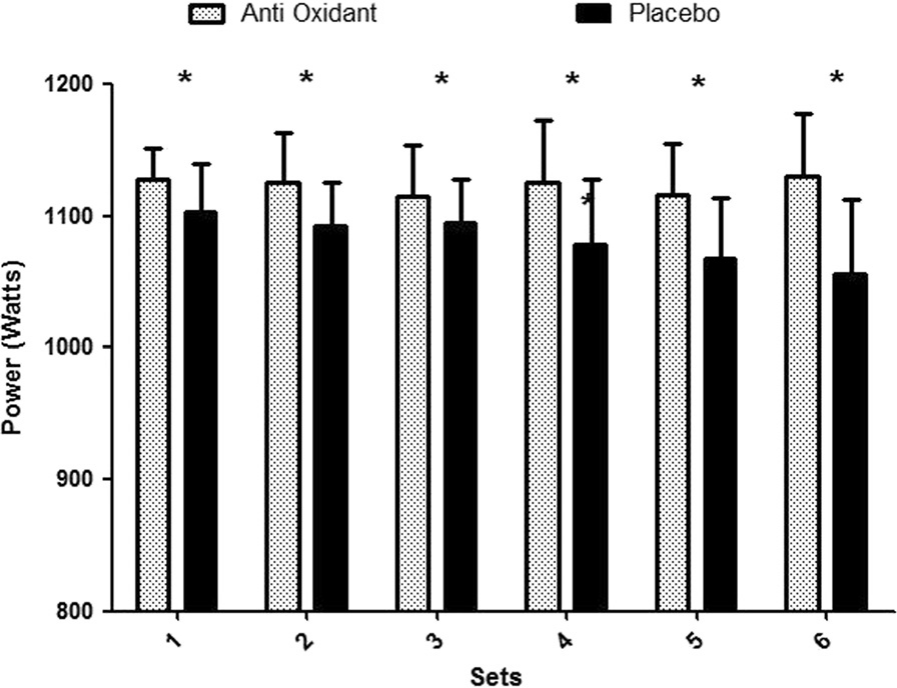
**Concentric power output for each set during the resistance training session (HTS) when AOX or placebo was ingested (mean ± SEM).** Statistically significant difference (*p < 0.05 and **p < .001) between the AOX and placebo trials.

**Figure 2:**
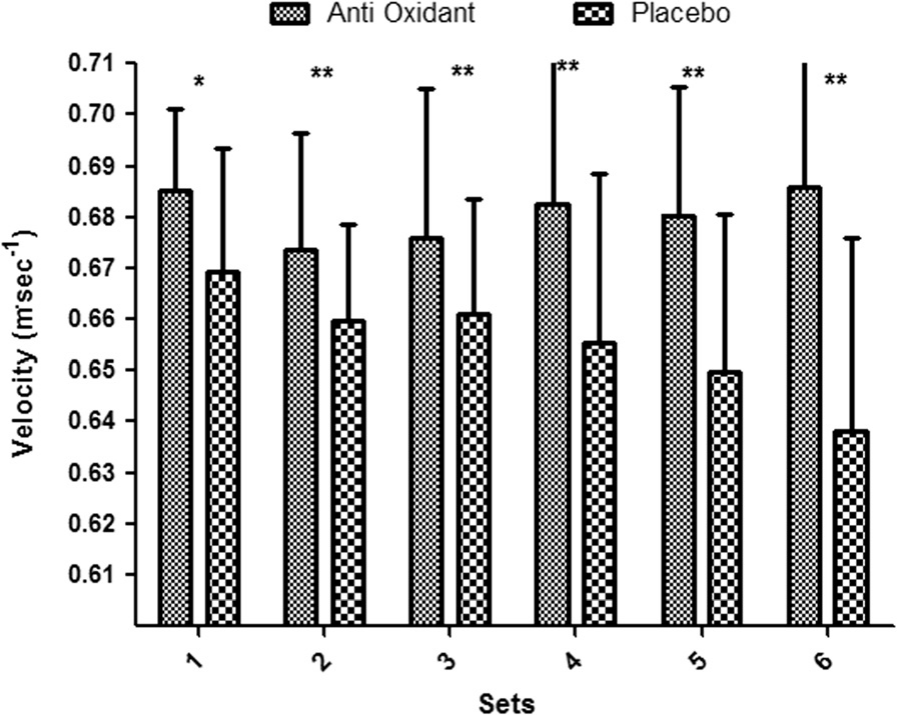
**Velocity (m.s) during each set of the resistance training session (HTS) when AOX or placebo was ingested (mean ± SEM).** Statistically significant difference (*p < 0.05 and **p < .001) between the AOX and placebo trials.

The concentric mean power and velocity are presented in Figures 1 and 2 respectively. Following AOX supplementation concentric mean power remained consistent across all six sets of the HTS. However, during the placebo trials concentric mean power significantly decreased from sets 1–6. During the placebo trial concentric mean power was significantly lower in comparison to each set in the AOX condition, with sets five and six having the greatest decrease (*p* < 0.05, ES’r = 0.52). Similarly average velocity during the AOX was higher compared to placebo. Accumulated power output during the AOX HTS was 6746 ± 5.9 W which was significantly greater compared to the placebo HTS of 6493 ± 17.1 W (*p* < 0.05, ES’r = 0.99).

**Figure 3:**
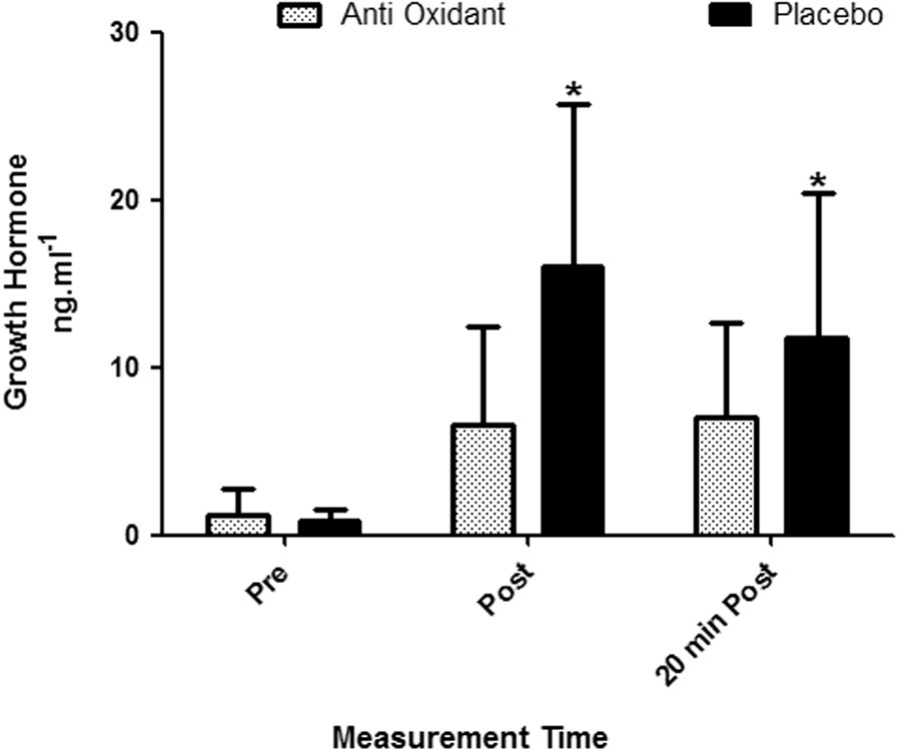
**Growth hormone (GH) in response to the AOX and placebo HTS (mean ± SEM).** Statistically significant difference (*p < 0.05 ) between the AOX and placebo trials.

The HTS resulted in a significantly elevated XO in both the placebo (pre: 11.05 ± 0.94 to immediately post: 15.47 ± 1.11 mU^.^ml^−1^) and AOX condition’s (pre: 9.16 ± 0.93 to immediately post: 11.2 ± 2.48 mU^.^ml^−1^, *p* < 0.05). The difference between the two conditions was not statistically significant (*p* > 0.05). Circulating GH levels increased significantly after both trials, however the increase was significantly less immediately following AOX supplementation; 6.65 ± 1.84 ng#x2219;ml^−1^ compared to the placebo trials;16.08 ± 2.78 ng#x2219;ml^−1^ (p < 0.05, ES’r = 0.89). GH continued to be significantly elevated 20 min after the HTS for both treatments, and was still significantly greater following the placebo trial in comparison to the AOX trial (*p* < 0.05) (Figure 3). Cortisol increased significantly immediately after the HTS following AOX and placebo supplementation to 567.25 ± 20.12 nmol#x2219;l^−1^ and 571.43 ± 18.77 nmol#x2219;l^−1^, respectively (*p* < 0.05). Cortisol was still significantly elevated 20 min post exercise for both treatments (*p* < 0.05) however there was no significant difference between the AOX and placebo HTS at any time point (*p* < 0.05).

## Discussion

The primary aim of the present research was to assess the effect of a PYC mixture on performance during lower limb ‘hypertrophic’ RT and the resulting acute endocrine, physiological and oxidative stress response. It was found that in comparison to a placebo mixture, subjects were able to perform 3.75% more work (W), and generate greater mean concentric power and velocity throughout the HTS after consuming the AOX mixture. An additional aim was to establish the physiological, endocrine and oxidative stress response to a HTS. There were no significant differences between RPE, B_lac_, CORT and XO between the two trials, however circulating GH levels was significantly reduced in the AOX trial compared to the placebo trial. This is the first study to demonstrate that an AOX mixture containing PYC can improve RT performance. There was a significant increase in B_lac_ levels immediately after both trials and 20 min post HTS from pre exercise values. The observed increase was similar to other RT protocols using high volume moderate loading intensity [36,37].

The attenuation of power decrement during the AOX trial agrees with previous studies that have shown that PYC AOX supplementation improves high intensity cycling and running performance [28,29]. This study was unique in that performance was monitored for each repetition and did not rely solely on the total volume for the session. Instead, concentric performance was measured by mean power output. The key finding from the performance data was that AOX supplementation was effective in attenuating the decrease in mean power which occurred in the placebo trial, meaning concentric power output was greater during the AOX trial (see Figure 1). During the placebo trial the mean power decrements per set ranged from 5% to 10% (specific data not shown). These observations are similar to the decrements observed by Baker and Newton [38], however their study employed a series of jump squats to elucidate a ROS response, and therefore comparisons between the two studies should be approached with caution.

The present study also found the oxidative stress response as measured by the marker XO was significantly increased after the HTS following both the placebo and AOX trials. This is similar to other studies which also observed an elevated XO response following strenuous exercise [13,39]. The significant rise in XO would suggest that the HTS in the present study invoked a substantial ROS response, which can lead to skeletal muscle injury and fatigue [1,39,40]. Indeed, reduced XO activity during RT has been linked to less oxidative damage and enhanced recovery from RT sessions [13]. It was therefore hypothesised that the AOX treatment would blunt the oxidative stress response, preserving skeletal muscle integrity and force production when performing strenuous RT such as BS exercise.

Yet, there was no significant difference in XO levels between the placebo and AOX trials, although a slight trend towards a reduction in XO following the AOX trials was observed (*p* = 0.069). There was also no difference in blood lactate concentration between the two conditions suggesting that differences in anaerobic fatigue were not the cause for the disparity in performance. This data suggests other mechanisms of muscular fatigue may have been involved in the performance changes observed. One possible mechanism is a decrease in Na+/K + ATPase pump activity [41]. A previous study found AOX supplementation in the form of N-acetyl-cysteine is effective in preserving Na+/K + ATPase activity during strenuous exercise, acting as a reduced thiol donor and promoting the regeneration of the endogenous AOX glutathione (GSH) [1,42]. Similarly, PYC supplementation has been shown to enhance GSH activity and decrease the levels of GSSG [43]. It is therefore possible that in the present study, the PYC based AOX supplement supported GSH levels which then lead to decreased thiol oxidation thus maintaining Na+/K + ATPase activity and attenuating muscular fatigue. Another possibility for the attenuation of muscular fatigue during the AOX trial may be a result of nitric oxide (NO) stimulated increased blood flow during exercise [44]. It has been repeatedly shown that PYC can enhance blood flow [23,25] and decrease platelet aggregation [45] which can decrease peripheral blood flow to contracting muscles during high intensity exercise [45]. At present it can only be speculated that these mechanisms were involved as GSH or muscular blood flow were not measured in this study. Further research with additional measures of oxidative stress is required to help determine the precise mechanisms involved in the performance improvements observed.

Cortisol increased significantly in both groups after the HTS and remained significantly elevated twenty min post exercise. However, there was no significant difference between the two groups at any time. Previous studies also found that similar RT protocols consisting of multiple set sessions with moderately high repetitions increases CORT secretion [34,46]. The catabolic activity of CORT may affect nitrogen balance after RT which in turn may hinder strength and/or MH development [47]. It would therefore be beneficial to attenuate CORT secretion during and after RT to avoid the deleterious effects that may interfere with training adaptations. At present, the effects of AOX supplementation on attenuating CORT and the underlying biochemical mechanisms involved is not well understood. Previous investigations with a similar design to the present study have produced mixed results. One study found positive results, where Vitamin C and E supplementation for 28 days significantly reduced post exercise increases in CORT following a lower body RT session. However, others agree with the present study, finding that an AOX treatment failed to mitigate the increase in CORT after a 90 min basketball training session [48] and a 90 min intermittent shuttle running protocol [49]. The discrepancy in results between the studies could be due to the type and duration of exercise sessions, and in particular the AOX supplementation type and dosage. Additional research should focus on using a greater dosage of PYC to further understand this compounds effects on CORT.

The GH response to the HTS was significantly affected by the AOX supplement. Immediately after the HTS the AOX group had a significantly lower GH response compared to the placebo group. This decreased circulating GH was also evident in the AOX group 20 min post exercise. This finding was unexpected as previous research showed PYC to be a potent secretagogue of GH in genetically engineered cells [26]. That the opposite occurred in this study is possibly related to the differing protocols and test subjects between the two, considering their findings were not observed in human subjects undertaking RT as in the present study. Another possible explanation is that GH secretion appears to be influenced by the degree of skeletal muscular fatigue induced by an exercise protocol. It has been demonstrated how increasing local muscular fatigue in the thigh by using forced exercise repetitions significantly increased GH response [50]. It’s therefore possible that during the placebo trials participants’ experienced greater levels of muscular fatigue, as evidenced by the reduced mean power output compared to the AOX trials, and thus leading to a greater GH response. Further research is needed to help determine this possibility and the potential role AOX supplementation has on GH secretion. Furthermore, as GH is an anabolic hormone its elevation during RT coupled with appropriate mechanical strain may be important for the process of muscular hypertrophy [51,52]. This would suggest that the GH results from this study indicate they may be undesirable in regards to promoting muscular hypertrophy. It is therefore of interest for future studies to examine whether this decreased circulating GH would affect muscular hypertrophy after a prolonged period of use or whether it acutely affects IGF-1 levels. Moreover, recent research suggests excessive AOX supplementation may hinder important physiological training adaptations [3,53]. This has prompted the suggestion that optimal oxidant content for maximal force production exists within the muscle [53]. These recent findings and the GH results in this study, highlight the need to further our understanding of the effect of AOX supplementation on training adaptations.

## Conclusions

In conclusion, an acute dose of a PYC based AOX supplement enhanced lower body RT performance in trained males by improving mean concentric power, velocity and total work output. The mechanisms involved are still unclear considering oxidative stress response (measured as plasma XO) was not significantly reduced in the AOX treatment, as hypothesised. Future studies should incorporate further measures of oxidative stress, particularly GSH, and muscle blood flow which may help determine the biochemical and physiological mechanisms that led to the results in this study. Furthermore, GH secretion was significantly attenuated in the AOX trial compared to the placebo. The mechanisms that led to these results are not fully understood, but further research is required as GH secretion is involved in MH and strength development and its attenuation may negatively impact training adaptations.

## Competing interests

The authors declare they have no competing interests.

## Authors’ contributions

DB and LRM conceived the concept for the investigation and contributed significantly to the drafting of the manuscript. JA was primary investigator in this study conducted the majority of testing and biochemical analysis. TC and DB assisted in data collection and provided a significant contribution to composition and review of the manuscript. All authors read and approved the final manuscript.
